# The association between living environmental factors and adolescents’ body weight: a cross-sectional study

**DOI:** 10.1186/s12887-021-03054-8

**Published:** 2021-12-13

**Authors:** Siyi Huang, Sha Sha, Wei Du, Hanwen Zhang, Xinyi Wu, Chongmin Jiang, Yan Zhao, Jie Yang

**Affiliations:** 1 Youth Sport Research & Development Center, China Institute of Sport Science, Beijing, China; 2 Department of Sport and Exercise Science, Zhejiang University, Hangzhou, China; 3 Australian National University College of Medicine Biology and Environment, Canberra, Australia; 4 Key Laboratory of Environmental Medicine Engineering, Ministry of Education; School of Public Health, Southeast University, Nanjing, China; 5 The First Affiliated Hospital, Sun Yat-sen University, Guangzhou, China; 6 School of Sports and Health, Nanjing Sport Institute, Nanjing, China; 7 Jiangsu Province CDC: Jiangsu Province Center for Disease Control and Prevention, Nanjing, China

**Keywords:** Excessive body weight, Living environmental factors, Adolescents

## Abstract

**Background:**

The effect of the living environment on public health has received increasingly scholarly attention. This study aims to explore the relationship between adolescents’ body weight and their living environmental factors.

**Methods:**

This cross-sectional study comprised 1362 middle-school students from Nanjing and 826 from Changzhou in China. We further collected information on living environmental factors based on their home address and ran multivariate logistic regressions to explore potential correlations after considering a range of potential confounding factors.

**Results:**

Approximately 25% (*n* = 303) of students from Nanjing and 26% (*n* = 205) of students from Changzhou were excessive body weight. In Nanjing, students’ BMI (Body Mass Index) showed a strong negative correlation with the number of sports venues in their neighborhood (Adjusted Odds Ratio (AOR): 0.64, 95%CI: 0.40–0.94) after controlling for other covariates. In Changzhou, we observed a positive correlation between adolescents’ body weight and the number of bus stops in their neighbourhood (AOR:1.63, 95%CI:1.11–2.38).

**Conclusions:**

The living environment factors were independently associated with teenagers’ excessive body weight. We hypothesis that the environmental risk factors might be associated with political management, which will consequently affect personal health outcomes. Further research and proactive measures are required to manage those potential risks and attenuate the problem.

**Supplementary Information:**

The online version contains supplementary material available at 10.1186/s12887-021-03054-8.

## Background

With the recent global urbanization and the continuing industrial development, the number of obese children has demonstrated a ten-fold increase from approximately 11 million in 1975 to 124 million in 2016, with additional 216 million overweight individuals worldwide [1]. Regretfully, over the recent three decades, little progress has been achieved in treating or preventing excessive body weight [2]. It was reported that in China, the prevalence of childhood obesity increased from 0.5% in 1985 to 7.3% in 2014, with a rising prevalence of overweight from 2.1 to 12.2% [3]. It is therefore obligatory for scholars to rethink the strategies for obesity and overweight prevention and treatment.

According to previous statistical analysis, overweight and obese individuals were divided into different groups, leading to underestimating the severity of the overweight issues, [4] especially common in low-income families [5]. Traditional policymakers and doctors [4] just paid attention to the severe obesity patients, ignoring health-care costs incurred by the overweight group. There were only a few overweight patients to link their terrible physiological situations with their excessive BMI [4, 6]. Overweight students and their parents, to some extent, did not realize that excessive body weight related to multiple adverse health outcomes, including an elevated risk of developing type 2 diabetes, cardiovascular diseases, and other physical and mental illnesses [7, 8]. The economic burden of overweight and obesity has also reached US$2 trillion, matching that of smoking and all military conflict [9]. Therefore, the whole range of excessive BMIs should be considered, as opposed to simply concentrating on the obesity group.

Considering that the individual genetic and lifestyle factors attribute in part to the global increase in the prevalence of obesity and overweight in recent years, the multifactorial nature of overweight has attracted much attention to researching on modifiable environmental characteristics [10, 11]. Living environmental factors influence students’ life behaviours and affect their energy intake and consumption, leading to different health outcomes. It is reported that an increased number of fitness facilities were associated with a reduction in teenagers’ body weight, which provided more opportunities to access to recreational facilities to increase their daily exercise [12, 13]. Pineda E et al. 2019 emphasized that the distance to the nearest fast-food restaurants elevated the risk of overweight and obesity, especially in low-family-income students [14]. A straightforward hypothesis is that the environmental characteristics, including the presence of cycle paths, sidewalks, active public transportation [15], green spaces [14] and the degree of urbanization, are related to childhood body weight. Access to open green spaces led to increased physical activities as well as decreased screen time which would perhaps explain the reduced risk of students’ excessive BMI [16–18].

Although the current research has addressed the significant relationship between environmental factors and adolescents’ excessive body weight, several essential aspects have not been scrutinized, such as parents’ attitudes towards students doing outdoor activities and influence from large-scale municipal works projects. Most studies on the relationship between the built environment and childhood excessive body weight, in addition, were mainly conducted in developed countries [19]. It was worthy to note that China, a nation with a highly authoritative government, has equipped with the fast-growing food delivery industry, the prosperity of sports marketing, full capacity running transportation system; this has resulted in different opportunities to access to healthy food or changing citizens’ life behaviours [19, 20]. However, to date, there has been little research systematically assessing the relationship between living environmental factors and childhood body weight in China, which is valuable for future environment design and city planning to reverse the tide of childhood excessive body weight.

Considering the foregoing, this study, focusing on the whole excessive body weight children, examines the living environmental factors in an urban Chinese context and evaluates how these living environmental factors relate to the risk of childhood excessive BMI. Findings may potentially contribute to the body of knowledge and inform the development of multi-sectoral intervention strategies.

## Methods

### Study design and participants

We conducted this population-based study in Nanjing and Changzhou, with a combined population of more than 1.5 million residents aged between 11 to 15 years old. These two cities have similar economic and cultural backgrounds, but Nanjing has a larger population and a more prosperous built environment setting than Changzhou. Therefore, we ran a parallel analysis for each city, respectively.

The cross-sectional survey was conducted in all districts in Changzhou and nine districts in Nanjing. We randomly selected one junior high school from each district, then chose three classes from each school and one class for each grade (i.e., Grade 7–9). We excluded residential students who lived in the school dormitories and those without any information on commuting to school, yielding a sample size of 1911 students from Nanjing and 1244 from Changzhou. We further excluded participants who did not provide complete data on home address, weight, and height. The final study population comprised 1362 students from Nanjing and 826 from Changzhou.

### Ethics

Before we conducted this survey, the written consent from participants and their parents were already obtained. Moreover, all information regarding the participants and their families remained confidential. The data were collected from June to October 2018, and this study was approved by China Institute of Sports Science ethics committee (Ethical code: CISSIRD-201604).

### Study outcome

According to the latest *State Students Health Standards*, we categorized BMI of the study participants into the normal weight group and excessive body weight group (combined overweight and obesity) [21]. We excluded 182 underweight students (127 from Nanjing, 55 from Changzhou) from the analysis. We ran sensitivity analysis and did not find material change.

### Study factors

To explore the impact of the domestic living environment on childhood excessive body weight, we used the Geography Information System (ArcGIS 9.1) to calculate the number of bus stops, scenic spots, sports venues, food spots, and recreational areas in 500 m distance from their home address. In this research, we defined the five aspects in advance.

1. bus stops mean the number of bus stops.
2. food spots, including restaurants, cafeterias, bubble tea stores, coffee shops, and street food spots.
3. scenic spots covering parks, historical places, and ancient temples.
4. sports venues including sports facilities center, public sports playgrounds, fitness trail, and stadium.
5. recreation areas covering shopping centers, zoos, museums, carnie, and aquaria.

We further categorized the number of bus stops as over 20 or not, scenic sites as over three or not, and the availability of sports venues and recreational areas as yes or no.

We also collected the controlling factors to modify the results, such as age, gender, daily physical activities, intake frequency of sweetened food and sugar beverage drink, parental BMI, parental smoking history, overall satisfaction with living environments, fitness time of parents themselves, family economic status, and so on, which might affect teenagers’ body weight.

### Measurements of physical activities

Based on the China health and nutrition survey questionnaire and Godin Shephard questionnaire, we designed our physical activity part in our questionnaire. The physical activity time consisted of exercise time at school, including physical education, recess, club activities, extracurricular activity time, and out of school, covering after-school interest classes, after-school playtime, and commuting time. Compendium of Energy Expenditures for Youth was used to identify the intensity of different exercises. We utilized reproducibility to check the reliability of the data. For the Moderate-to-Vigorous Physical Activities (MVPA) at school, we gained the students’ schooling timetable from the educational staff. At the same time, students also reported their spending time in MVPA at school. Parents and children respectively reported spending time for exercise out of school. Reproducibility was assessed via intra-class correlation coefficients for both spending time at and out of school (Intraclass Correlation Coefficient, ICC > 0.7), so the data was considered reliable.

### Measurements of diet behaviours

Due to our pilot study, it was not easy for teenagers to accurately recall the types and sizes of food they ate in 48 h. Therefore, we collected the frequency of high sweetened food or fried food in the diet aspect rather than daily energy consumption.

### Statistical analysis

We employed Stata 14.0 for all data analysis. The number and proportion of variables of interest among the sampled population were calculated. We conducted multiple imputation for missing values before running a univariate logistic analysis for each environmental variable and the outcome to explore the strength of the association. Environmental variables of the *p*-value of less than 0.25 for the crude odds ratio (OR) were modelled with multivariate logistic regression for each city respectively, to control for other covariates. Priori confounders included age, sex, and other sociodemographic variables. We used the likelihood ratio test to test if the difference between the full model and the reduced model was statistically significant. We also used mixed-effect logistic models for each city for sensitivity analysis to examine if regional (district-level) variabilities would contribute to the childhood excessive body weight. We observed little clustering effects with negligible intraclass coefficients. We considered a *p*-value of 0.05 as statistically significant.

## Results

Excessive body weight was more prevalent in males than females (31% vs 18% in Nanjing, 31% vs 20% in Changzhou) (Tables 1 and 2). The prevalence of excessive body weight increased with the frequency of consuming barbeque and fried food among Nanjing students (Table 1), which was not observed among Changzhou students (Table 2).

**Table 1:** Study population characteristics, by Body Mass Index – normal vs overweight, Nanjing

Variables	BMI			Univariate Analysis	
	*Normal*	*Excessive bodyweight*	*Total*		
	No. (%)	No. (%)	No. (%)	*Crude OR [95%CI]*	*P*
Age					
≤ 12	232 (77)	69 (23)	301 (24.4)	–	
13	333 (72)	127 (28)	460 (37.2)	1.28 [0.91,1.8]	0.15
14	244 (79)	66 (21)	310 (25.1)	0.91 [0.62,1.33]	0.63
≥ 15	123 (75)	41 (25)	164 (13.3)	1.12 [0.72,1.75]	0.62
Sex					
Female	514 (82)	113 (18)	627 (50.8)	–	
Male	418 (69)	190 (31)	608 (49.2)	**2.07 [1.58,2.7]**	**< 0.0001**
***Dietary and Exercise factors***					
Moderate-to-vigorous physical activity					
≤ 30 min/d	600 (78)	172 (22)	772 (62.5)	–	
> 30 min/d	332 (72)	131 (28)	463 (37.5)	**1.38 [1.06,1.79]**	**0.018**
Sweeten food				1.02 [0.91,1.15]	0.72
Never	232 (75)	76 (25)	308 (24.9)		
1–2 times/month	278 (77)	83 (23)	361 (29.2)		
1–2 times/week	250 (74)	89 (26)	339 (27.4)		
2–3 times/week	114 (75)	39 (25)	153 (12.4)		
Every day	36 (77)	11 (23)	47 (3.8)		
Unknown	22 (81)	5 (19)	27 (2.2)		
BBQ and fried food				**1.19 [1.04,1.36]**	**0.012**
Never	389 (78)	109 (22)	498 (40.3)		
1–2 times/month	358 (75)	117 (25)	475 (38.5)		
1–2 times/week	106 (71)	43 (29)	149 (12.1)		
2–3 times/week	46 (70)	20 (30)	66 (5.3)		
Every day	10 (59)	7 (41)	17 (1.4)		
Unknown	23 (77)	7 (23)	30 (2.4)		
Snacks				0.98 [0.89,1.09]	0.77
Never	188 (73)	68 (27)	256 (20.7)		
1–2 times/month	213 (76)	66 (24)	279 (22.6)		
1–2 times/week	256 (77)	78 (23)	334 (27)		
2–3 times/week	148 (75)	50 (25)	198 (16)		
Every day	96 (75)	32 (25)	128 (10.4)		
Unknown	31 (78)	9 (23)	40 (3.2)		
***Family factors***					
Economic status				**0.76 [0.6,0.97]**	**0.03**
Lower	409 (73)	148 (27)	557 (45.1)		
Medium	407 (78)	117 (22)	524 (42.4)		
Higher	33 (87)	5 (13)	38 (3.1)		
Unknown	83 (72)	33 (28)	116 (9.4)		
Father’s BMI					
Normal	517 (80)	131 (20)	648 (52.5)	–	
Overweight	271 (68)	126 (32)	397 (32.1)	**1.93 [1.34,2.79]**	**< 0.0001**
Underweight	31 (79)	8 (21)	39 (3.2)	0.78 [0.46,1.31]	0.96
Unknown	113 (75)	38 (25)	151 (12.2)		
Mother’s BMI					
Normal	643 (77)	187 (23)	830 (67.2)	–	
Overweight	98 (64)	55 (36)	153 (12.4)	**1.83 [1.38,2.44]**	**< 0.0001**
Underweight	84 (82)	19 (18)	103 (8.3)	1.02 [0.46,2.27]	0.35
Unknown	107 (72)	42 (28)	149 (12.1)		0.13
Parents’ encouragement for exercise					
Less encourage	473 (74)	162 (26)	635 (51.4)	–	
More encourage	401 (77)	120 (23)	521 (42.2)	0.88 [0.67,1.17]	0.38
Unknown	58 (73)	21 (27)	79 (6.4)		
Father fitness time					
Shorter	469 (76)	145 (24)	614 (49.7)	–	
Longer	273 (75)	90 (25)	363 (29.4)	1.02 [0.76,1.36]	0.92
Unknown	190 (74)	68 (26)	258 (20.9)		
Mother fitness time					
Shorter	459 (76)	146 (24)	605 (49)	–	
Longer	391 (75)	133 (25)	524 (42.4)	1.05 [0.8,1.37]	0.74
Unknown	82 (77)	24 (23)	106 (8.6)		
Father smoking					
No	346 (80)	87 (20)	433 (35.1)	–	
Yes	552 (73)	202 (27)	754 (61.1)	**1.46 [1.09,1.94]**	**0.01**
Unknown	34 (71)	14 (29)	48 (3.9)		
Mother smoking					
No	858 (76)	270 (24)	1128 (91.3)	–	
Yes	8 (67)	4 (33)	12 (1)	**1.59 [0.47,5.32]**	0.45
Unknown	66 (69)	29 (31)	95 (7.7)		
Satisfaction towards living environment					
Less satisfied	165 (73)	61 (27)	226 (18.3)	–	
More satisfied	740 (76)	231 (24)	971 (78.6)	0.85 [0.61,1.18]	0.33
Unknown	27 (71)	11 (29)	38 (3.1)		
Parents’ satisfaction towards living environment					
Less satisfied	159 (75)	54 (25)	213 (17.2)	–	
More satisfied	710 (76)	220 (24)	930 (75.3)	0.88 [0.64,1.23]	0.46
Unknown	63 (68)	29 (32)	92 (7.4)		
***Environmental factors***					
Bus stops					
≤ 20	88 (76)	28 (24)	116 (9.4)	–	
> 20	844 (75)	275 (25)	1119 (90.6)	0.98 [0.62,1.53]	0.92
Scenic spots					
0–3	236 (76)	73 (24)	309 (25)	–	
> 3	696 (75)	230 (25)	926 (75)	1.07 [0.79,1.44]	0.67
Sport venues					
No	97 (68)	46 (32)	143 (11.6)	–	
Yes	835 (76)	257 (24)	1092 (88.4)	**0.65 [0.44,0.95]**	**0.025**
Casual spots					
No	84 (81)	20 (19)	104 (8.4)	–	
Yes	848 (75)	283 (25)	1131 (91.6)	1.4 [0.85,2.32]	0.19
Food spots (median)	198 [0,2087]	216 [0,2179]	207 [0,2179]	1 [1,1]	0.27
**Total**	**932 (75)**	**303 (25)**	**1235**		

*OR* Odds ratio, *p p*-value

**Table 2:** Study population characteristics, by Body Mass Index – normal vs overweight, Changzhou

Variables	BMI			Univariate Analysis	
	*Normal*	*Excessive bodyweight*	*Total*		
	No. (%)	No. (%)	No. (%)	*Crude OR [95%CI]*	*P*
Age					
≤ 13	139 (74)	49 (26)	188 (23.6)	–	
14	420 (74)	146 (26)	566 (71)	0.99 [0.68,1.44]	0.94
≥ 15	33 (77)	10 (23)	43 (5.4)	0.86 [0.39,1.87]	0.70
Sex					
Female	326 (80)	84 (20)	410 (51.4)	–	
Male	266 (69)	121 (31)	387 (48.6)	**1.77 [1.28,2.44]**	**0.001**
***Dietary and Exercise factors***					
Moderate-to-vigorous physical activity					
≤ 30 min/d	185 (75)	62 (25)	247 (31)	–	
> 30 min/d	407 (74)	143 (26)	550 (69)	1.05 [0.74,1.48]	0.79
Sweeten food				1.03 [0.89,1.2]	0.65
Never	172 (75)	57 (25)	229 (28.7)		
1–2 times/month	165 (77)	49 (23)	214 (26.9)		
1–2 times/week	147 (68)	68 (32)	215 (27)		
2–3 times/week	68 (83)	14 (17)	82 (10.3)		
Every day	18 (69)	8 (31)	26 (3.3)		
Unknown	22 (71)	9 (29)	31 (3.9)		
BBQ and fried food				1.1 [0.92,1.32]	0.3
Never	264 (78)	76 (22)	340 (42.7)		
1–2 times/month	205 (71)	85 (29)	290 (36.4)		
1–2 times/week	69 (73)	26 (27)	95 (11.9)		
2–3 times/week	27 (82)	6 (18)	33 (4.1)		
Every day	4 (57)	3 (43)	7 (0.9)		
Unknown	23 (72)	9 (28)	32 (4)		
Snacks				0.97 [0.85,1.11]	0.67
never	154 (73)	57 (27)	211 (26.5)		
1–2 times/month	144 (74)	51 (26)	195 (24.5)		
1–2 times/week	154 (77)	45 (23)	199 (25)		
2–3 times/week	77 (72)	30 (28)	107 (13.4)		
Every day	41 (76)	13 (24)	54 (6.8)		
Unknown	22 (71)	9 (29)	31 (3.9)		
***Family factors***					
Economic status				0.89 [0.67,1.19]	0.44
Lower	199 (73)	75 (27)	274 (34.4)		
Medium	260 (75)	85 (25)	345 (43.3)		
Higher	30 (79)	8 (21)	38 (4.8)		
Unknown	103 (74)	37 (26)	140 (17.6)		
Father’s BMI					
Normal	315 (78)	87 (22)	402 (50.4)	–	
Overweight	193 (67)	93 (33)	286 (35.9)	**1.74 [1.24,2.46]**	**0.001**
Underweight	21 (88)	3 (13)	24 (3)	0.52 [0.15,1.77]	0.30
Unknown	63 (74)	22 (26)	85 (10.7)		
Mother’s BMI					
Normal	418 (79)	110 (21)	528 (66.2)	–	
Overweight	60 (53)	54 (47)	114 (14.3)	**3.42 [2.24,5.22]**	**< 0.0001**
Underweight	63 (79)	17 (21)	80 (10)	1.03 [0.58,1.82]	0.932
Unknown	51 (68)	24 (32)	75 (9.4)		
Parents’ encouragement for exercise					
Less encourage	324 (75)	108 (25)	432 (54.2)	–	
More encourage	200 (72)	76 (28)	276 (34.6)	1.1 [0.79,1.55]	0.57
Unknown	68 (76)	21 (24)	89 (11.2)		
Father fitness time					
Less	286 (74)	102 (26)	388 (48.7)	–	
More	265 (74)	91 (26)	356 (44.7)	0.97 [0.7,1.35]	0.86
Unknown	41 (77)	12 (23)	53 (6.6)		
Mother fitness time					
Less	306 (74)	110 (26)	416 (52.2)	–	
More	235 (76)	75 (24)	310 (38.9)	0.91 [0.65,1.26]	0.57
Unknown	51 (72)	20 (28)	71 (8.9)		
Father’s history of smoking					
No	240 (75)	82 (25)	322 (55.3)	–	
Yes	325 (74)	116 (26)	441 (40.4)	1.04 [0.75,1.45]	0.79
Unknown	27 (79)	7 (21)	34 (4.3)		
Mother’s history of smoking					
No	563 (74)	193 (26)	756 (0.8)	–	
Yes	6 (100)	0 (0)	6 (94.9)	N/A	
Unknown	23 (66)	12 (34)	35 (4.4)		
Students’ satisfaction towards living environment					
Less satisfied	37 (64)	21 (36)	58 (7.3)	–	
More satisfied	548 (75)	180 (25)	728 (91.3)	0.59 [0.34,1.03]	0.06
Unknown	7 (64)	4 (36)	11 (1.4)		
Parents’ satisfaction towards living environment					
less satisfied	52 (74)	18 (26)	70 (8.8)	–	
More satisfied	477 (73)	173 (27)	650 (81.6)	1.04 [0.6,1.8]	0.88
Unknown	63 (82)	14 (18)	77 (9.7)		
***Environmental factors***					
Bus stops					
≤ 20	189 (80)	48 (20)	237 (29.7)	–	
> 20	403 (72)	157 (28)	560 (70.3)	**1.53 [1.06,2.21]**	**0.022**
Scenic spots					
0–3	43 (81)	10 (19)	53 (6.6)	–	
> 3	549 (74)	195 (26)	744 (93.4)	1.53 [0.75,3.1]	0.241
Sport venues					
No	1 (100)	0 (0)	1 (0.1)	–	
Yes	591 (74)	205 (26)	796 (99.9)	N/A	
Casual spots					
No	4 (67)	2 (33)	6 (0.8)	–	
Yes	588 (74)	203 (26)	791 (99.2)	0.69 [0.13,3.8]	0.67
Food spots (median)	362 [22,1068]	372 [22,1031]	362 [22,1068]	1 [1,1]	0.88
**Total**	**592 (74)**	**205 (26)**	**797**		

*OR* Odds ratio, *p p*-value

### Study site 1: Nanjing

More students had parents with normal BMI range (52.5%, *n* = 648 among fathers; 67.2%, *n* = 830 among mothers), less parental encouragement for exercise (51.4% *n* = 635), a father with smoking history (61.1%, *n* = 754), and were satisfied with their living environment (78.6%, *n* = 971 among students; 75.3%, *n* = 930 among their parents). Students lived in a residential address with over 20 bus stops (90.6%, *n* = 1119), over 3 scenic spots (75%, *n* = 926), with any sport venues (88.4%, *n* = 1092) and any recreational areas (91.6%, *n* = 1131) within 500-m distance, accounted for a large majority of the study population. The median number of food outlets was 207 with a range from 0 to 2179.

Compared with students residing in a place without any sports venues, those who had access to sports venues near their residential places are less likely to gain excessive body weight (AOR: 0.64, 95%CI: 0.40–0.94, *P* = 0.027), after controlling for other factors (Table 3). In addition to the sport venues, male students (AOR: 2.01, 95%CI: 1.49–2.71, *P* < 0.0001), those having an excessive BMI parent (AOR: 1.87, 95%CI: 1.38–2.52, *P* < 0.0001 for father; AOR: 1.78, 95%CI: 1.20–2.62, *P* = 0.004 for mother), from lower socioeconomic background (AOR: 0.70, 95%CI: 0.52–0.94, *P* = 0.017), having higher frequency of barbeque and fried food consumption (AOR: 1.18, 95%CI: 1.01–1.38, *P* = 0.037), and having medium strength activity of longer than 30 min (AOR: 1.40, 95%CI:1.04–1.90, *P* = 0.025) were more likely to gain excessive body weight.

**Table 3:** Adjusted odds ratios for characteristics against adolescents’ overweight, Nanjing – Results from Reduced Model

Adjusted OR – Reduced Model		
	AOR	*P*
**Age**		
≤ **12**	–	
13	1.30 [0.88,1.93]	0.193
14	0.93 [0.61,1.44]	0.749
15	1.04 [0.61,1.78]	0.877
**Sex**		
Females	–	
Males	**2.01 [1.49,2.71]**	**< 0.0001**
**Sport venues**		
No	–	
Yes	**0.61 [0.40,0.94]**	**0.027**
**Father’s BMI**		
Normal	–	
Underweight	0.94 [0.39,2.23]	0.882
Overweight	**1.87 [1.38,2.52]**	**< 0.0001**
**Mother’s BMI**		
Normal	–	
Underweight	0.89 [0.51,1.54]	0.672
Overweight	**1.78 [1.20,2.62]**	**0.004**
**Economic status**	**0.70 [0.52,0.94]**	**0.017**
**BBQ & Fried food**	**1.18 [1.01,1.38]**	**0.037**
**Moderate-to-vigorous physical activity**		
≤ 30 min	–	
> 30 min	**1.40 [1.04,1.90]**	**0.025**

*AOR* Adjusted odds ratio, *p p*-value

### Study site 2: Changzhou

In contrast, more students had parents with normal BMI range, less parental encouragement for exercise (54.2% *n* = 432), fathers without smoking history (55.3%, *n* = 322), and were satisfied with their living environment (91.3%, *n* = 728 for students themselves; 81.6%, *n* = 650 for their parents). Students lived in a residential address with over 20 bus stops (70.3%, *n* = 560) and over 3 scenic spots (93.4%, *n* = 744) accounted for the majority of the study population. Only six students resided in a place without any recreational areas. The median number of food outlets was 362 with a range from 22 to 1068.

Univariate analysis identified that those were males, either having an excessive BMI parent or residing in a place with over 20 bus stops within 500-m distance, were positively associated with being excessive body weight (*P* < 0.05) (Table 2). The effects of these factors remained after controlling for other covariates (Table 4). Using multilevel mixed-effect models, we did not observe statistically significant regional variations at district levels (intraclass correlation < 0.01, *P* > 0.05). In Table 4, we also observed a positive correlation between adolescents’ body weight and the number of bus stops in their neighbourhood (AOR:1.63, 95%CI:1.11–2.38).

**Table 4:** Adjusted odds ratios for characteristics against adolescents’ overweight, Changzhou– Results from Reduced Model

Adjusted OR - Reduced model		
	OR [95%CI]	*P*
**Age**		
≤ 13	–	
14	0.92 [0.62,1.36]	0.67
15	0.95 [0.42,2.11]	0.91
**Sex**		
Females	–	
Males	**1.78 [1.27,2.48]**	**0.001**
**Bus**		
≤ 20	–	
> 20	**1.63 [1.11,2.38]**	**0.011**
**Mother’s BMI**		
Normal	–	
Underweight	1.03 [0.57,1.84]	0.93
Overweight	**3.57 [2.32, 5.50]**	**< 0.0001**

*AOR* Adjusted odds ratio, *p p*-value

## Discussion

In this geographically diverse sampling of adolescents from junior high schools, our results illustrated consistent evidence that closing to a sports venue and numbers of near bus stops were independently correlated with adolescents’ body weight. To be specific, the number of bus stops put a negative impact on the prevention of the excess body weight among Changzhou students, while the high availability of sports venues created a positive effect for Nanjing students, which was in line with the development of city policies and guidelines years [22]. Nanjing was the host city for the 2nd Summer Youth Olympic Game in 2014, and the local administrations were dedicated to developing the best global sports and entertainment venues and enhancing the sports culture. After the Game, its heritage was transferred for public activities, and the culture prevailed [23]. According to the 2016 government report, additional efforts would be invested to mend fitness trails to grow from 420 km to 863 km, provide 691 playgrounds, and establish 341 sports facilities during the period from 2017 to 2035 [23]. Lacking comparable sports venues and culture, Changzhou is famous for its well-organized public transportation system with low bus fare, expanding public transportation network, and high daily passenger capacity [24]. However, our findings were contrary to the previous results that more number of bus stops would lead to a high prevalence of childhood excessive body weight [25]. The convenient public transportation system did not show a positive impact on individuals’ travelling habits. However, it is beyond the capacity of this brief research to fully contextualize this complex issue and further qualitative research is needed to explore the hidden reasons for this weird phenomenon.

Mendenhall argued that macro-level political elements would influence chronic diseases on their clustering at the population level and consequently would affect syndrome pathologies at the individual level [26]. This implies that decisive policies can reverse the upwards trend of excessive body weight by modifying civil planning and access to facilities, especially for vulnerable younger children with constrained resources [27]. The young excessive body weight relates to merely eating and exercising habits and encompasses essential aspects of social and environmental situations, which might exacerbate their health outcomes and inequity [27, 28]. Facing with limited access to resources due in part to family financial burden and personal study load, provision of free sports facilities, ideally near residential neighbourhood, might be sufficient to offer extra opportunities for adolescents to attain the unstructured exercise during their off-school time [29]. Additional exercise is necessary to finally make up the time of daily active exercise to catch up with the recommended levels of physical activities, which is currently not met in school-aged adolescents by large [30].

It is noteworthy that the Changzhou subway project was conducted in April 2015, and line 1 was finished in Sep 2019; the completion of line 2 will be at the beginning of 2021. In the former investments, researchers indicated the subway construction process was the “predawn darkness” for the traffic system, and the long period of metro construction would bring numerous environmental and social issues for this city [31]. Usually, subways lines have coincided with the urban traffic-intensive hubs. It was inevitable that the construction enclosure occupied the parts of the crossroad and motorway and damaged some traffic lights, decreasing the surrounding safety [32]. Subway construction posed a substantial extra burden on surface transportation, causing traffic congestion. Chaos traffic surroundings set barriers for citizens to access to public transportation, which might influence their health behaviours towards more active lifestyles, with the implication of more likely to be excessive body weight [32]. Based on local government 2018 reports, the car ownership per capita of Nanjing and Changzhou was almost the same, 0.25 and 0.24 per capita respectively [33]. It was also noteworthy the nearly perfect public transportation system did not create the walkability of residential surroundings and decrease personal car-dependence.

In addition to the built environment factors, other factors, including parental BMI, family income, being male, and barbeque food consumption, were associated with young adolescents’ excessive body weight, consistent with previous findings [14, 34–36]. For example, genetic factors influenced fat distribution as well as daily energy expenditure, energy intake, and habitual physical activities [14]. It is also speculated that families with higher incomes could have more access to healthy food and live healthier lifestyles [37]. However, the lack of comparable exposure data limited our ability to confirm such relationships. Besides, there was a positive correlation between moderate physical activity and gaining excessive body weight in our project. Previous reports have demonstrated that acute aerobic exercise could generate a short-term energy deficit, disturbing the personal energy balance and contributing to a biological inevitability postexercise compensatory eating and energy intake [38, 39]. It was noticed that the extent of compensation could be different among individuals [40]. To some populations, the compensatory adjustment might increase individual energy intake, but some could not. Whereas, considering the financial budget and operationalization of the experiment, we did not collect the information on the daily food types and sizes that students ate, which need further exploration.

Excessive body weight in school-aged children in China remains a public health issue. Further efforts to reduce childhood excessive body weight are warranted [41]. Future studies using trial data or investigation of individual and parental beliefs and behaviours can further explore the role of environmental factors and the effects of local policies in particular availability of sports venues and access to the public transportation network concerning childhood excessive body weight.

### Strength and limitations

This cross-sectional study assessed the environmental factors of excessive body weight with descriptive data and a relatively sizeable random sample comprising 1362 students from Nanjing and 826 from Changzhou. Our study compared two cities located on the southeast coast of China: Nanjing and Changzhou. Both cities are equipped with a similar economic and cultural background but differ significantly in political status, which resulted in differences in policy implementation. Nanjing is the capital city of Jiangsu province, so the annual financial expenditure and policy implementation will be skewed towards it. For the first time, we found the potential impacts of policy decisions on local adolescents’ body weight.

What is more, we also boldly attempted to incorporate obese and overweight individuals into the excessive body weight group to avoid underestimating the severity of the overweight group. The high level of BMI has already brought a significant burden on their family and society. We thus have to understand that the purpose of our interventions was to decrease the mean of BMI the whole excessive body weight rather than decline the number of the severely obese population [4].

However, this cross-sectional study suffers several limitations, including the lack of crime data (for security reasons, we did not gain the data from police departments) concerning young adolescents’ residential areas. We consequently were not able to explore whether satisfaction with neighbourhood safety would impact young adolescents’ physical activities [42]. Besides, financially we did not utilise the ActiGraph GT3X-BT accelerometer to collect participants’ daily energy consumption data. The inaccuracy of total diet energy intake by the self-report was documented in the former researches, and the accuracy of energy intake did not meet our requirements in our pilot study [43]. We, therefore, chose the alternative variables, such as the frequency of sweetening food and high-fat food. Moreover, the nature of cross-sectional data limits the cause-effect inference because we assessed the exposure and outcomes simultaneously, and it was difficult for us to tell whether the outcome followed exposure in time. Further qualitative research is needed to explore the motivations and hidden reasons for personal behaviours.

## Conclusions

In conclusion, this study indicates that the living environment factors were independently associated with excessive body weight. The findings among students from Nanjing and Changzhou varied due to different local policies. Therefore, we hypothesis that, to some extent, the environmental risk factors, e.g., numbers of bus stations and sports venues, might be associated with political management, which will finally affect individual behaviours and personal health outcomes. Given that teenagers’ excessive body weight is still a significant health concern, further research and proactive measures are required to attenuate the problem.

## Supplementary Information

**Additional file 1.**

## Data Availability

Datasets used and/or analysed during the current study are available from the corresponding author on reasonable request.

## Abbreviations

AOR: Adjusted Odds Ratio
BMI: Body Mass Index
ICC: Intraclass Correlation Coefficient
MVPA: Moderate-to-Vigorous Physical Activities
OR: Odds Ratio
