# Supplementary figures and images for: The association between living environmental factors and adolescents’ body weight: a cross-sectional study
Source: BMC Pediatr. 2021 Dec 13;21:572. doi: 10.1186/s12887-021-03054-8 (PMC8667439; doi:10.1186/s12887-021-03054-8)

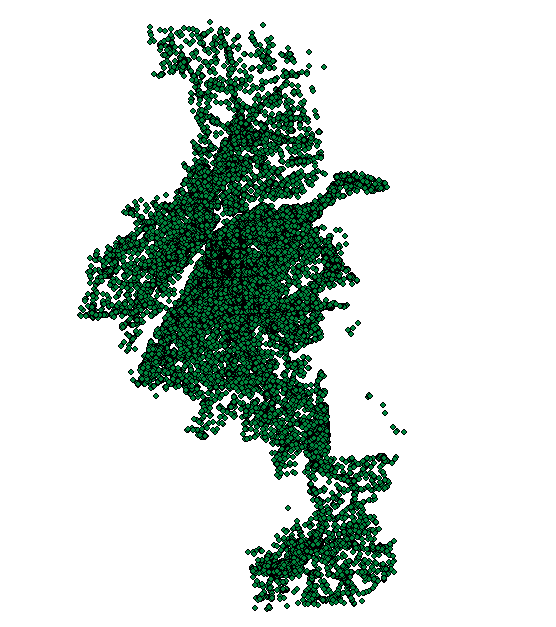

Supplement: Supplementary file 1 — Additional file 1. [file 12887_2021_3054_MOESM1_ESM.png]
